# Estimated dietary iodine intake as a predictor of placental size: evidence from the ELSPAC study

**DOI:** 10.1186/s12986-018-0240-8

**Published:** 2018-01-17

**Authors:** Julie Bienertová-Vašků, Markéta Grulichová, Ondřej Mikeš, Filip Zlámal, Tomáš Pruša, Aneta Pohořalá, Lenka Andrýsková, Hynek Pikhart

**Affiliations:** 1 Research Centre for Toxic Compounds in the Environment (RECETOX), Faculty of Science, Masaryk University, Kamenice 5, building A29, 625 00 Brno, Czech Republic; 2 Department of Pathological Physiology, Faculty of Medicine, Masaryk University, Kamenice 5, building A18, 625 00 Brno, Czech Republic; 3 Research Department of Epidemiology and Public Health, University College London, 1-19 Torrington Place, London, WC1E 6BT UK

**Keywords:** Iodine intake, Placental weight, Birth weight / placental weight, Birth outcome, ELSPAC

## Abstract

**Background:**

The relationship between low iodine status and pregnancy-associated comorbidities has been known for decades. The relationship between iodine intake and placental pathologies is, however, far less clear. This study was designed to examine the relationship between dietary iodine intake and placental size while also focusing on typical adverse pregnancy outcomes.

**Method:**

The dietary iodine intake of 4711 pregnant women enrolled in the Czech part of the European Longitudinal Study of Pregnancy and Childhood (ELSPAC) in 1990–1991 was established using a 145-item food frequency questionnaire. Multivariate linear regression models were used to estimate the relationship between dietary iodine intake during pregnancy and placental weight. Additional models were constructed to investigate the relationship between estimated dietary iodine intake and adverse birth outcomes.

**Results:**

The estimated average iodine intake in the ELSPAC cohort was 106.6 μg/day. In the fully adjusted model, estimated dietary iodine intake was found to be significantly negatively associated with placental weight (β = −0.025, 95% CI: -0.044; −0.006, *p* = 0.011). Moreover, estimated dietary iodine intake was found to be significantly positively associated with the birth weight / placental weight ratio in the fully adjusted model (β = −0.024, 95% CI: 0.004; 0.043, *p* = 0.016).

**Conclusions:**

This study provides evidence of a relationship between estimated dietary iodine intake and placental weight and the birth weight / placental weight ratio. Additional research is warranted to provide more insight into the role of iodine in early as well as late placentation.

## Background

Iodine is an essential micronutrient necessary for the synthesis of thyroid hormones. It has long been recognized that foetal as well as obstetric outcomes are dependent on normal maternal thyroid function [1]. Severe iodine deficiency during foetal development results in maternal and foetal hypothyroidism and is associated with serious adverse health effects, including mainly cretinism and prenatal as well as postnatal growth retardation [1]. As confirmed many times, thyroid hormones are crucial for the correct development of the nervous system of the child [2].

Physiologically, gestation calls for significant alterations in the functioning of the thyroid system, with increased maternal iodine requirements consequently reaching over 50% [3, 4]. This increase is associated with increasing foetal demand on thyroid hormones synthesized by the mother. While the foetus is capable of synthesizing its own thyroid hormones beginning with the 16th to 20th week of gestation, during the 8th to 10th week the foetus utilizes exclusively maternal hormones and is thus fully dependent on maternal synthesis at this very early gestation stage [4–6].

Human chorionic gonadotropin (hCG), a pregnancy-associated hormone, is involved in the stimulation of the thyrotropin receptor (TSH-receptor), resulting in the consequent increase in the level of the circulating hormone leading to suppressed TSH levels via negative feedback. In addition to hCG, factors directly influencing maternal thyroid function include i) increased renal iodine clearance, ii) estrogen-dependent increase in thyroxine-binding globulin and iii) placental deiodinase activity. The establishment of cut-off values for thyroid hormones in pregnancy/lactation therefore requires an approach which takes into account gestational age. Among the above mentioned factors, increasing iodine clearance is of considerable importance in pregnancy as renal plasma flow increases by 75% in mid-pregnancy and there is a 50% higher glomerular filtration rate (GFR) between the first and third trimesters [7]. The increased renal clearance of iodine can therefore lead to relatively lower plasma inorganic iodide (PII) concentration, which in turn leads to higher sodium-iodide symporter activity in the thyroid. This situation could then lead to increased thyroid iodide clearance in order to compensate for the relative changes of plasmatic iodide and mainly to keep the absolute uptake of iodide within the same range [7].

As iodine is deeply involved in the development and maintenance of a healthy pregnancy, pregnant women and their foetuses as well as infants are most vulnerable to its deficiency. However, there is no unanimous consensus on the issue of iodine supplementation in pregnancy. In 2007, the WHO/UNICEF/ICCIDD [8] reached a limited consensus, stipulating that iodine-containing supplements should not be recommended to pregnant women if a given population has been iodine sufficient for at least two years. More specifically, if the population in general has an adequate iodine intake with the median urinary iodine concentration (UIC) ≥100 μg/l but lower than 150 μg/l, both the American Thyroid Association [9] and the European Thyroid Association [10] recommend supplementation. This approach, however, is not currently advocated by the WHO, which recommends an iodine intake of 250 μg/day during pregnancy, with salt iodization listed as the key strategy suitable for fighting possible deficiencies.

According to the WHO, the Czech Republic now ranks among countries where iodine deficiency is not considered a major public health issue. However, the Central European region has a long history of moderate to severe iodine deficiency. Salt iodisation was first introduced in Czechoslovakia (now the Czech Republic) in 1947, with a subsequent dramatic decrease in cases of severe iodine deficiency [11]. Median urinary iodine values in general as well as in hospitalized adult populations shifted, with initial values indicating mild iodine deficiency (88–95 μl/l) prior to 1997, reaching a critical threshold of 100 μl/l in 1998 and achieving optimal values of 120–140 μl/l by 2000 [11]. Between 1995 and 2016, urinary iodine concentration monitoring was conducted in various population groups, with the most recent study (2015) observing median urinary iodine concentrations in pregnant Czech women to be 151 μl/l while approximately 50% of studied women fulfilled criteria of iodine deficiency [12].

While sufficient information on urine iodine levels is available, information on dietary iodine intake in the Czech Republic and its association with public health risk is very scarce. This study therefore aims to evaluate the estimated dietary iodine intake in pregnancy in a large prospective cohort from the European Longitudinal Study of Pregnancy and Childhood (ELSPAC), contextualize the achieved results with respect to dietary iodine and investigate the relationship between iodine intake and defined birth outcomes.

## Methods

### Study subjects

ELSPAC is a population-based study designed to investigate environmental and other factors affecting the health and development of children from the prenatal period until adulthood [13]. The study was initiated as one of six prospective birth cohort studies by the WHO Regional Office for Europe in 1985 and was designed to accommodate a total of 40,000 children across Europe [14]. The project was initially coordinated by Bristol University (Avon Longitudinal Study of Pregnancy and Childhood; ALSPAC [15]) and included eight independent centres based in the United Kingdom, Czechoslovakia, Ukraine, Greece and Russia. In the Czech part of the study, all eligible mothers were living in Brno or Znojmo (cities in Czechoslovakia) and were expected to deliver between 1 April 1991 and 30 June 1992. A total of 4711 participants who answered over 50% of the selected questions were included in the analyses. Approximately 9.7% of all answers were missing and were imputed, as described further. A total of 67 women who were on thyroid hormone replacement or anti-thyroid drugs or who reported thyroid disease were excluded from the study.

### Ethics statement

Ethical approval for the study was obtained from the European Longitudinal Study of Pregnancy and Childhood (ELSPAC) Law and Ethics committee and local research ethics committees. Written informed consent was obtained from all study participants and archived.

### Dietary assessment

Participating women were asked to answer two questionnaires during pregnancy, a questionnaire about themselves (P1 – About You, Mother) and a questionnaire about their pregnancy (P2 – I am Expecting a Baby). A self-reported food frequency questionnaire (FFQ) was included as part of the P2 questionnaire package in accordance with the ELSPAC protocol [14] at 32 weeks of gestation in 1991–1992. Based on available domestic and foreign sources as well as on an expert assessment of portion sizes carried out by nutrition specialists, a list of individual food items associated with specific portion sizes which also reflected the composition of food items in the early 1990s was developed. The final questionnaire included 145 items in 36 separate food groups. The utilization of domestic sources allowed us to define portion sizes not only for global foods but also for local or regional foods, in accordance with an approach previously used e.g. by the HAPIEE study [16]. Answers to a total of 44 questions from the P2 questionnaire were used to calculate iodine intake. The results of iodine content analysis for individual items and food groups are provided in Table 1.

**Table 1:** Estimated iodine content in selected items/groups

List of food groups	Food item	Iodine content [μg/100 g or 100 ml]
Refined flour bakery products	bread	4.0
	rolls	
	French bread	
Whole-wheat bakery products	graham bread	4.0
	whole grain roll	
Breakfast cereals	oatmeal	4.0
	puffed rice	
	popcorn	
	porridge	11.0
	flavoured granola	
	bran	1.3
	grain sprouts	
Kolaches, sweet rolls	sweet rolls	4.9
	kolaches	
	cakes	
	soufflés	
	leavened cakes	
	sponge cake	
	doughnuts	8.9
	desserts	
	omelettes	
Biscuits, wafers, sponge biscuits	sponge biscuits	13.5
	filled wafers	
Puddings	fruit-based	14.1
	milk-based	
	fruit yoghurt	
	whipped cottage cheese products	
Poultry	chicken	1.1
	ducks	
	goose	
Red meat	beef	3.1
	pork	
	mutton	
	ham	
	bacon	
Pâtés	pâtés	7.5
	minced meat	
Offal	liver	11.9
	kidney	
	heart	
Sausages	smoked meat	7.8
	frankfurters	
	sausages	
	other smoked meat products	
	hamburgers	
	hamburger patties	
Pizza		6.5
Fish	canned fish	30.0
	fresh fish	25.0
	frozen fish	
Shellfish	oysters	116.0
	crabs	
	snails	
Eggs	eggs	50.0
	breakfast eggs	50.0
Cheese	cheese	7.0
	breakfast cheese	7.0
Legumes	baked beans	4.0
	peas	5.8
	lentils	
	soybeans	
	breakfast beans	4.3
	breakfast soybeans	
Nuts	nuts	5.8
	roasted nuts	
	salted nuts	
Fried potatoes	French fries	3.6
Roasted potatoes		9.0
Boiled potatoes	boiled potatoes	9.3
	mashed potatoes	
Pasta		9.0
Rice	cooked rice	3.0
Leaf vegetables	cabbage	4.8
	kale (leaf cabbage)	
	cauliflower	
	spinach	
	leek	
	green beans	
Root vegetables	carrot	7.9
	parsley	
	celery	
	beet	
Salads	lettuce	3.4
	cucumber salad	
	tomato salad	
Fresh fruit	apples	3.2
	pears	
	oranges	
	bananas	
	grapes	
	plums	
Fruit juices		0.8
Cola	Cola	0.5
	Pepsi	
Tea except for herbal tea		0.1
Coffee		0.5
Herbal teas		0.2
Chocolate	chocolate	14.5
	confectionery	
Milk	milk	14.4
	dairy products	
Honey		2.0
Soft drinks	lemonade	3.2
	soda	

Food estimates were combined with iodine content data from national databases and reported as μg of iodine per 100 g or 100 ml. To achieve a precise and complete evaluation of iodine intake from all possible sources, the following databases were used in hierarchical order: Czech Food Composition Database [17], Slovak Online Food Database [18], National Food Composition Database in Finland [19], Danish Food Composition Databank [20], Composition of Foods Integrated Dataset (CoFID) [21] and the Nutridan nutritional programme database [22]. The consumption coefficient was derived from retrospective food consumption data based on databases from 1920 to 2006. The average portion was defined for all food item categories with respect to definitions in view of the fact that portion sizes have changed throughout the post-communist period, that weights and volumes of many foods have changed over time and that current sizes thus cannot be used. Generally, portion sizes were derived from two domestic sources from the 1990s: the Verbal and Graphic Formulation of Dietary Guidelines for the Czech Republic project [23] and the Czech National Food Consumption Survey, implemented in the 2003–2006 period [24]. A weighted average of portion sizes for the entire food group was then calculated based on the consumption ratio of individual foods.

### Birth outcomes

Selected birth outcomes were treated as dependent variables in the models: birth length and weight, placental weight and cephalisation and ponderal indices. Gestational age, gestational diabetes, gestational hypertension, stillbirth, congenital malformations, pre-eclampsia and IVF pregnancy were included as independent variables. All data were collected by healthcare professionals at the maternity hospital at the time of birth. Gestational age was determined using ultrasound dating.

Pre-eclampsia was defined in accordance with the International Society for the Study of Hypertension in Pregnancy (ISSHP) criteria [25] as systolic blood pressure ≥ 140 mmHg and/or diastolic blood pressure ≥ 90 mmHg along with proteinuria of ≥1+ on urine dipstick testing occurring on two occasions after 20 weeks of gestation. Small for gestational age was defined as being below the 10th percentile of birth weight adjusted for gestational age at birth using internal standardisation by cohort. Preterm birth was defined as birth before 37 weeks of gestation. Gestational diabetes was defined as any record of a diagnosis of gestational diabetes at any time during the pregnancy in women without existing diabetes at the beginning of pregnancy.

Birth weight was measured to a precision of 0.1 kg. Placental weight was reported by the doctor present at birth. All possible gestational complications were summarised in the physician’s birth questionnaire (N1). The ponderal index was calculated as birth weight (kg) divided by birth length cubed (m^3^). The cephalisation index was expressed as the ratio of head circumference at birth (cm × 10^4^) to birth weight (g) and later expressed as μm/g [26].

### Covariates

Several variables may be considered to constitute potential confounding factors for the investigated birth outcomes. In order to construct statistical models for defined birth outcomes, the following independent variables were included in the analyses, as appropriate: dichotomised comorbidities in present and previous pregnancies (gestational diabetes at present, pre-eclampsia, gestational hypertension associated with a current pregnancy, stillbirth occurring in the past, in vitro fertilization associated with a current pregnancy and congenital malformations in the past), gestational age, maternal pre-conceptional weight, maternal age at birth, maternal smoking prior to pregnancy (never smoker, smoker in the past, smoker in the last nine months), living with partner status (yes–no), self-reported alcohol intake during pregnancy and self-reported drug abuse during pregnancy. Maternal socioeconomic status, a variable generally associated with adverse birth outcomes, was assessed based on several variables: maternal education (categorized according to ISCED), maternal job (categorized according to ISCO 88 and ISCO 08 into 16 groups and 4 skill levels), ethnicity (Caucasian, other) and family income calculated according to Eurostat recommendations for equalized family income [27], and appropriately adjusted to. Since extreme maternal height may sometimes be associated with an increased risk of adverse birth outcomes, all models were adjusted to also accommodate maternal height. Moreover, as advanced maternal age as well as offspring gender may be associated with worse obstetric outcomes, adjustments taking into account maternal age at birth as well as child gender were also made.

### Statistical modelling

Dietary iodine intake was analysed using 44 food items with established iodine content selected from FFQs administered to study subjects. Uniformity was tested by examining the distribution of variables in a loading plot verified using the Kaiser-Meyer-Olkin (KMO) measurement of adequacy with a value of 0.5 considered as acceptable for further analysis. The Bartlett sphericity test was used to establish whether the correlation matrix differed significantly from the identity matrix [28]. Orthogonal varimax rotation was used to minimize the number of indicators which might have high loading on one factor [29].

The relationship between maternal dietary iodine intake and birth outcomes was investigated using linear regression models. Multivariate models were built for each birth outcome separately in two steps. First, the association between maternal dietary iodine intake and birth outcomes was investigated using a crude unadjusted analysis using the univariate models. Second, biological factors (maternal height, paternal height, maternal preconception weight and maternal age), social and demographic characteristics (maternal and paternal education, ethnicity, occupation, equalized family income), maternal health and comorbidities of the mother and child (gestational diabetes, gestational hypertension, stillbirth, child congenital malformations, IVF pregnancy and pre-eclampsia), maternal alcohol consumption, substance abuse, smoking and use of nutritional supplements during pregnancy, and finally gestational age and sex of the child were included as potential confounders into final (adjusted) multivariate models.

Missing data were imputed using the Multiple Imputation by Chained Equations method [30] and regression models were built using imputed data. All analyses were performed using R, version 3.1.2 and STATA, version 14. *P*-values of less than 0.05 were considered statistically significant.

## Results

### Population characteristics

The basic anthropometric and demographic characteristics of the study subjects are provided in Table 2.

**Table 2:** Basic anthropometric/demographic characteristics of the study subjects

variable	unit	mean	sd	median	5th perc.	95th perc.	min	max
Iodine intake	μg/day	106.6	42.4	101.1	49.2	175.6	6.7	612.5
Family income	CZK/month	5490	2490	5000	2600	9000	1500	48,000
Equalized family size	person	2.2	0.6	2.1	1.8	3.3	1.0	10.5
Equivalized family income	CZK/month	2596	1286	2478	1071	4444	364	26,667
Maternal age	y	25.2	4.8	24.6	18.8	34.7	15.2	48.7
Gestational age	week	39.7	1.7	39.9	36.7	41.9	23.1	44.6
Birth length	cm	50.2	2.3	50.0	46.0	53.0	30.0	59.0
Birth weight	g	3290	493	3300	2500	4050	600	5350
Placental weight	g	587	164	560	380	860	115	1500
Head circumference	cm	34.6	1.4	35.0	32.0	37.0	23.0	43.0
Cephalisation index	μm/g	107.2	17.6	104.7	87.7	133.3	60.7	500.0
Ponderal index	kg/m^3^	25.9	2.3	25.8	22.5	29.6	16.3	49.3
Birth weight / Placental weight	–	5.9	1.6	5.8	3.7	8.9	1.5	17.3
Maternal height	cm	166.3	6.0	167.0	157.0	176.0	135.0	189.0
Paternal height	cm	179.6	6.8	180.0	169.0	191.0	152.0	204.0
Maternal preconc. weight	kg	61.0	9.9	60.0	48.0	80.0	38.0	135.0
Maternal preconc. BMI	kg/m^2^	22.0	3.3	21.5	18.1	28.3	15.1	46.1
Energy intake	kJ/day	6558	2157	6272	3563	10,273	2504	19,362
FS Prudent	–	0.0	1.0	−0.1	−1.4	1.9	−2.1	3.9
FS Carbohydrates	–	0.0	1.0	−0.1	−1.5	1.8	−3.1	3.5
FS Junk food	–	0.0	1.0	−0.1	−1.5	1.8	−2.8	3.7

### Dietary patterns

Food group analysis was performed using factor analysis. The adequacy of data for analysis was evaluated based on the KMO value [31]; the result of Bartlett’s sphericity test (χ^2^ = 14,435, df = 630, *p* < 0.001) provided strong statistical evidence that the correlation matrix was significantly different from the identity matrix.

Three factors identified in pregnant women explained 14.6% of total variance. The first factor – which accounted for 6.2% of total variance among mothers – was labelled the prudent factor. This factor may be characterized by high factor loading in the case of whole-wheat bakery products, fish, legumes, fresh vegetables, root vegetables and rice. The second factor – which accounted for 4.5% of total variance – was labelled the carbohydrate–dairy factor and included foods such as refined flour bakery products, pudding, cheese, fresh fruit, milk and dairy products. The third factor – which accounted for 3.9% of total variance – was labelled the junk food factor and included items such as biscuits, wafers, pies and cakes, French fries, roasted potatoes, pâté and ground meat. The factors scores of the individual patterns have been reported in our previous paper focusing on the association of anthropometry with dietary patterns of mothers in the third trimester [32].

### Estimated iodine intake and birth outcomes

In order to address the potential association between estimated iodine intake and placental size in the context of birth outcomes, we first performed an estimation of average dietary iodine intake by assigning average iodine intake to individual food items. Since no longitudinal comprehensive Czech database providing measured iodine levels for a broad spectrum of foods in the relevant period (1991–1992) is currently available, we derived iodine intake data from multiple available databases and systematically contextualized iodine content with individual FFQ items. As considerable differences in reported iodine intake between databases from various geographical regions were established, we based our methodology on the origin of iodine in foods in close geographical regions. For example, while the Czech Food Composition Database [17] reported values of iodine content in fish to be 0–4.1 μg/100 g, the National Food Composition Database in Finland [33] reported a value of 105 μg/100 g and the Danish Food Composition Databank [34] reported a value of 253 μg/100 g. In such cases, we used values from the database from a geographical region closest to the Czech Republic. While the highest iodine content was reported for infant formula, powdered soups, smoked fish, canned meat and sausages, milk and saltwater fish, the most frequently consumed and thus most important dietary sources included milk, baked goods, eggs, saltwater fish, potatoes, pasta and yoghurt [35]. The high dietary exposure to iodine from animal sources is the result of massive iodine supplementation of animal feed in the case of animals destined for meat and egg production, ongoing throughout the study period.

Once various birth outcomes included in the initial model were reduced to dependent variables, we obtained a single final model with a statistically significant relationship between estimated iodine intake and placental weight. We observed a negative association which indicates that every 100 μg of daily iodine intake during pregnancy is significantly associated with a 2.5% decrease in placental weight (95% CI: 0.6;4.3%).

Quartiles of cohort subjects with respect to estimated iodine intake are provided in Table 3. Models for other outcomes including e.g. birth weight were non-significant and are not discussed further. The results of univariate as well as multivariate regression modelling for the logarithm of placental weight are provided in Table 4. The table shows the estimated coefficients of linear regression in unadjusted (univariate) and fully adjusted (multivariate) models for the effects of maternal dietary iodine intake and other covariates on placental weight. Results with other birth outcomes as dependent variables are not shown as no significant associations with dietary iodine were observed. In the final model for the natural logarithm of placental weight, dietary estimated iodine intake was found to be a significant predictor of logarithm of placental weight (β = −0.025, 95% CI: -0.044; −0.006, *p* = 0.011).

**Table 3:** Quartiles of study subjects in relation to birth outcomes and estimated iodine intake (as mean ± sd)

Variable	Estimated iodine intake [μg/day]				
	Q1 (6.6; 79.7]	Q2 (79.7; 101.0]	Q3 (101.0; 127.5]	Q4 (127.5; 612.5]	*P*- value
Birth length [cm]	50.1 ± 2.3	50.2 ± 2.2	50.3 ± 2.3	50.1 ± 2.3	0.112
Birth weight [g]	3288 ± 470	3287 ± 470	3325 ± 493^a^	3263 ± 501^a^	0.035
Placental weight [g]	591 ± 168	595 ± 167^a^	587 ± 166	576 ± 163^a^	0.035
Cephalisation index [μm/g]	107.5 ± 20.1	107.1 ± 16.4	106.0 ± 16.8^a^	108.0 ± 17.3^a^	0.059
Ponderal index [kg/m^3^]	25.9 ± 2.2	25.9 ± 2.5	26.0 ± 2.3	25.8 ± 2.2	0.266
Birth weight/Placental weight [−]	5.9 ± 1.6	5.9 ± 1.6	6.0 ± 1.55	6.0 ± 1.6	0.083

^a^significant differences between means in post-hoc analysis using Tukey-Kramer method

**Table 4:** The effects of iodine intake and other covariates on natural logarithm of placental weight in univariate and multivariate models

	Univariate models			Multivariate model		
	β estimate	95% CI for β estimate	*p*-value	β estimate	95% CI for β estimate	*p*-value
Iodine intake [100 μg/day]	−0.032	(−0.050; −0.013)	<0.001	−0.025	(−0.044; −0.006)	0.011
Gestational diabetes						
No	(ref)			(ref)		
Yes	−0.088	(−0.149; −0.027)	0.005	−0.080	(−0.140; −0.020)	0.009
Pre-eclampsia						
No	(ref)			(ref)		
Yes	−0.105	(−0.208; −0.002)	0.046	−0.133	(−0.234; −0.031)	0.011
Gestational hypertension						
No	(ref)			(ref)		
Yes	0.062	(0.010; 0.113)	0.019	0.053	(0.001; 0.104)	0.046
Gestational age [w]	0.015	(0.010; 0.019)	<0.001	0.013	(0.009; 0.018)	<0.001
Stillborn						
No	(ref)			(ref)		
Yes	−0.064	(−0.149; 0.022)	0.143	−0.056	(−0.140; 0.028)	0.193
IVF pregnancy						
No	(ref)			(ref)		
Yes	0.131	(0.041; 0.220)	0.004	0.141	(0.051; 0.230)	0.002
Congenital malformations						
No	(ref)			(ref)		
Yes	0.016	(−0.025; 0.057)	0.453	0.016	(−0.025; 0.056)	0.443
Smoking						
Never	(ref)			(ref)		
Past smoker	0.012	(−0.005; 0.029)	0.152	0.008	(−0.009; 0.025)	0.353
Current smoker	−0.024	(−0.052; 0.005)	0.100	−0.019	(−0.049; 0.011)	0.206
Substance abuse						
No	(ref)			(ref)		
Yes	−0.050	(−0.134; 0.034)	0.239	−0.033	(−0.116; 0.051)	0.443
Alcohol						
No	(ref)			(ref)		
Yes	−0.018	(−0.048; 0.013)	0.261	−0.020	(−0.050; 0.011)	0.213
Nutritional supplements						
No	(ref)			(ref)		
Yes	0.050	(0.035; 0.066)	<0.001	0.047	(0.031; 0.063)	<0.001
ISCO skill level - mother						
Skill level 1	(ref)			(ref)		
Skill level 2	0.005	(−0.031; 0.040)	0.805	0.001	(−0.036; 0.037)	0.978
Skill level 3	−0.009	(−0.048; 0.030)	0.639	−0.015	(−0.056; 0.027)	0.493
Skill level 4	−0.006	(−0.043; 0.031)	0.741	−0.014	(−0.055; 0.027)	0.506
Skill level unassigned	−0.051	(−0.176; 0.075)	0.428	−0.056	(−0.179; 0.068)	0.377
ISCO skill level - father						
Skill level 1	(ref)			(ref)		
Skill level 2	−0.006	(−0.059; 0.046)	0.814	−0.007	(−0.059; 0.044)	0.783
Skill level 3	−0.026	(−0.083; 0.031)	0.369	−0.019	(−0.076; 0.038)	0.508
Skill level 4	−0.013	(−0.066; 0.041)	0.641	0.010	(−0.046; 0.065)	0.734
Skill level unassigned	0.004	(−0.056; 0.064)	0.894	0.026	(−0.033; 0.085)	0.386
Education – mother						
Basic	(ref)			(ref)		
Secondary	0.005	(−0.012; 0.023)	0.529	0.022	(0.002; 0.043)	0.035
University and higher	0.000	(−0.021; 0.022)	0.977	0.033	(0.001; 0.065)	0.043
Education – father						
Basic	(ref)			(ref)		
Secondary	−0.001	(−0.021; 0.018)	0.880	−0.001	(−0.023; 0.021)	0.936
University and higher	−0.016	(−0.035; 0.003)	0.090	−0.025	(−0.054; 0.004)	0.091
Equalized family intake [1000 CZK/month]	−0.002	(−0.011; 0.008)	0.698	−0.002	(−0.012; 0.009)	0.732
Age - mother [y]	0.001	(−0.001; 0.002)	0.377	0.000	(−0.001; 0.002)	0.669
Height - mother [cm]	0.002	(0.000; 0.003)	0.012	0.002	(0.000; 0.003)	0.014
Height - father [cm]	0.000	(−0.001; 0.001)	0.973	0.000	(−0.002; 0.001)	0.501
Maternal preconceptional BMI [kg/m^2^]	0.008	(0.006; 0.010)	<0.001	0.007	(0.005; 0.010)	<0.001
Ethnicity – mother						
European	(ref)			(ref)		
Others	−0.029	(−0.100; 0.042)	0.429	0.020	(−0.063; 0.104)	0.632
Ethnicity – father						
European	(ref)			(ref)		
Others	−0.039	(−0.094; 0.016)	0.168	−0.018	(−0.083; 0.047)	0.591
Sex of the child						
Girl	(ref)			(ref)		
Boy	0.029	(0.014; 0.045)	<0.001	0.029	(0.014; 0.045)	<0.001

### Estimated iodine intake and birth weight / placental weight ratio

The results of univariate as well as multivariate regression modelling for the logarithm of birth weight / placental weight ratio are provided in Table 5.

**Table 5:** The effects of iodine intake and other covariates on natural logarithm of birth weight/placental weight ratio in univariate and multivariate models

	Univariate models			Multivariate model		
	β estimate	95% CI for β estimate	*p*-value	β estimate	95% CI for β estimate	*p*-value
Iodine intake [100 μg/day]	0.015	(−0.005; 0.035)	0.136	0.024	(0.004; 0.043)	0.016
Gestational diabetes						
No	(ref)			(ref)		
Yes	0.093	(0.030; 0.156)	0.004	0.106	(0.045; 0.167)	<0.001
Pre-eclampsia						
No	(ref)			(ref)		
Yes	0.008	(−0.100; 0.116)	0.883	0.039	(−0.065; 0.143)	0.460
Gestational hypertension						
No	(ref)			(ref)		
Yes	−0.099	(−0.153; −0.045)	<0.001	−0.085	(−0.138; −0.032)	0.002
Gestational age [w]	0.042	(0.037; 0.046)	<0.001	0.041	(0.036; 0.046)	<0.001
Stillborn						
No	(ref)			(ref)		
Yes	−0.008	(−0.097; 0.081)	0.860	0.008	(−0.076; 0.092)	0.848
IVF pregnancy						
No	(ref)			(ref)		
Yes	−0.116	(−0.210; −0.023)	0.015	−0.126	(−0.216; −0.037)	0.006
Congenital malformations						
No	(ref)			(ref)		
Yes	−0.029	(−0.071; 0.013)	0.179	−0.035	(−0.075; 0.006)	0.095
Smoking						
Never	(ref)			(ref)		
Past smoker	−0.012	(−0.029; 0.005)	0.178	−0.012	(−0.029; 0.005)	0.172
Current smoker	−0.031	(−0.060; −0.002)	0.039	−0.019	(−0.049; 0.011)	0.215
Substance abuse						
No	(ref)			(ref)		
Yes	−0.029	(−0.113; 0.056)	0.501	0.000	(−0.083; 0.083)	0.998
Alcohol						
No	(ref)			(ref)		
Yes	0.015	(−0.018; 0.048)	0.372	0.016	(−0.015; 0.046)	0.317
Nutritional supplements						
No	(ref)			(ref)		
Yes	−0.050	(−0.066; −0.034)	<0.001	−0.045	(−0.061; −0.028)	<0.001
ISCO skill level - mother						
Skill level 1	(ref)			(ref)		
Skill level 2	0.009	(−0.028; 0.047)	0.634	0.006	(−0.031; 0.042)	0.764
Skill level 3	0.029	(−0.011; 0.070)	0.160	0.016	(−0.026; 0.058)	0.444
Skill level 4	0.039	(0.000; 0.078)	0.047	0.019	(−0.022; 0.061)	0.364
Skill level unassigned	0.018	(−0.111; 0.147)	0.786	0.031	(−0.093; 0.155)	0.621
ISCO skill level - father						
Skill level 1	(ref)			(ref)		
Skill level 2	0.021	(−0.034; 0.076)	0.449	0.010	(−0.043; 0.063)	0.709
Skill level 3	0.037	(−0.023; 0.097)	0.225	0.007	(−0.052; 0.067)	0.816
Skill level 4	0.039	(−0.016; 0.095)	0.162	−0.012	(−0.069; 0.045)	0.688
Skill level unassigned	−0.027	(−0.088; 0.033)	0.378	−0.038	(−0.097; 0.021)	0.206
Education – mother						
Basic	(ref)			(ref)		
Secondary	0.013	(−0.005; 0.030)	0.164	−0.019	(−0.040; 0.002)	0.081
University and higher	0.030	(0.008; 0.053)	0.009	−0.029	(−0.062; 0.004)	0.081
Education – father						
Basic	(ref)			(ref)		
Secondary	0.017	(−0.003; 0.037)	0.092	0.009	(−0.014; 0.031)	0.444
University and higher	0.036	(0.016; 0.055)	<0.001	0.030	(0.000; 0.060)	0.047
Equalized family intake [1000 CZK/month]	0.009	(−0.001; 0.019)	0.080	0.005	(−0.006; 0.016)	0.382
Age - mother [y]	0.002	(0.000; 0.003)	0.048	0.001	(−0.001; 0.003)	0.463
Height - mother [cm]	0.003	(0.002; 0.004)	<0.001	0.001	(0.000; 0.003)	0.059
Height - father [cm]	0.002	(0.001; 0.004)	<0.001	0.001	(0.000; 0.003)	0.028
Maternal preconceptional BMI [kg/m^2^]	−0.001	(−0.004; 0.001)	0.238	−0.001	(−0.004; 0.001)	0.345
Ethnicity – mother						
European	(ref)			(ref)		
Others	−0.075	(−0.150; −0.001)	0.048	−0.012	(−0.098; 0.073)	0.780
Ethnicity – father						
European	(ref)			(ref)		
Others	−0.037	(−0.094; 0.020)	0.204	0.011	(−0.054; 0.075)	0.740
Sex of the child						
Girl	(ref)			(ref)		
Boy	0.016	(0.000; 0.032)	0.051	0.014	(−0.001; 0.029)	0.076

## Discussion

Multiple physiological changes in thyroid function take place during pregnancy; aberrant thyroid function during pregnancy has been associated with adverse obstetric outcomes [36]. Since the adverse impact of thyroid dysfunction on foetal development and obstetric outcomes has been empirically observed for decades, the issue of whether to screen for maternal thyroid dysfunction antenatally or preconceptionally or not is subject to an ongoing debate which has yet to be resolved in a satisfactory way. Furthermore, supplementation guidelines are also being debated worldwide. However, insufficient information is currently available on the role of iodine dietary intake on birth outcomes and subsequent child development, especially as most studies focus on the effects of supplementation and not on modified dietary composition. Exceptions include studies by Bath et al. [37], Hynes et al. [38] and Abel et al. [39], which report that even a mild degree of iodine deficiency in pregnancy is associated with verbal IQ, reading accuracy and reading comprehension at 8 years [37], educational outcomes at 9 years [38] and child language delay, behavioural problems and reduced fine motor skills at 3 years of age [39].

Based on our results, iodine intake may potentially change the weight of the placenta or the birth weight / placental weight ratio by several percent. This supports a hypothesis which states that the placenta may serve to some extent as an iodine reservoir capable of expanding in size to increase its absorption potential in the case of lower dietary iodine intake, i.e. in a manner similar to the thyroid gland. The same association of iodine intake and placental size has been observed in mice [40]. Based on empirical observations of the detrimental effects of inadequate or excessive dietary iodine intake during pregnancy for foetal development and/or birth outcomes both for the mother and the child, it can be speculated that only a narrow range of optimum iodine levels leads to optimum outcomes. An existing animal study has reported the linear association of increasing toxicity with increasing dietary iodine intake and the association of decreasing placenta size with the linear growth of dietary iodine [40], which thus supports the hypothesis that maternal thyroid hormones are directly involved in placental growth and development control.

The above mentioned findings are in line with the current understanding of the role of the trophoblast in the regulation of iodine and thyroid hormone transfer from the mother to the foetus. While the foetal thyroid starts to concentrate iodine after the first 10–12 weeks of gestation, the synthesis and secretion of the thyroid hormone under TSH control starts at approximately 20 weeks of gestation [41]. Prior to the beginning of the autonomous synthesis of thyroid hormones, the foetus fully relies on maternal thyroxine and utilizes both T3 and T4 to stimulate EGF production by primary cytotrophoblasts, thus promoting their survival [42]. Transplacental transport of maternal thyroid hormones is supported by the evidence of biologically significant concentrations of free thyroxine (t4) in foetal coelomic fluid, while these concentrations are highly independent on the thyroid of the offspring (in foetuses with absent thyroid, up to 50% of normal concentrations of t4 can be observed) [43].

The transplacental passage of thyroid hormones from mother to foetus and the following supply of the thyroid hormones into trophoblast cells requires the expression of placental plasma membrane transporters for thyroid hormones. The human placenta displays a wide range of transporters for thyroid hormones from early gestation: MCT8, MCT10, LAT1, LAT2, OATP1A2 and OATP4A1; the observed expression patterns are different between pregnancies with normal physiology and pregnancies affected with intrauterine growth restriction [44]. These transporters have been recently shown to accumulate iodine in the placental cell lines and are generally upregulated by hCG [44].

The delivery of the maternal thyroid hormones to the foetus therefore depends on i) the availability of the thyroid hormones in the maternal circulation and ii) the functional effectiveness of the transplacental transport systems. Current knowledge of thyroid hormone transporters not only strongly suggests that the placenta plays a role in iodine uptake, but also supports the hypothesis that the placenta may act as a storage capacity supplying iodine to the foetus gradually [45]. This is supported by recently established evidence which indicates that placental iodine uptake is saturable, which is typical of the thyroid gland [46]. To conclude, while the thyroid has a demonstrably greater capacity for iodide update than the placenta, the placenta and thyroid appear to share numerous iodine uptake and efflux mechanisms [45].

The exact mechanism and reason for decreasing placental size with increasing dietary iodine is, however, unknown and deserves further investigation. Although the importance of aberrant placental size is generally unclear, some evidence indicates that placental size may be associated with future developmental/cardiometabolic conditions. Some studies suggest that placental size is correlated with birth weight as well as with placental function [47]. Under certain conditions, e.g. malnutrition, the placental surface may increase in order to obtain more nutrients for the foetus, which may in turn result in increased placental weight at birth [48, 49]. On the whole, while a small placenta has previously been associated with premature birth risk, increased placental size has been associated with an increased rate of adverse conditions in newborns [50]. Some studies also associate placental size and weight with cardiometabolic risks later in life, e.g. with the risk of hypertension [51] or with total bone mass [52]. The association of placental size with bone mass and density is of special interest, as it was identified in ALSPAC, the mirror cohort to ELSPAC. However, any interpretation of such results between various ethnic groups and populations is complicated [53] and requires further investigation.

The gold standard for the estimation of iodine status in the general population today is urinary iodine concentration measurement [54]. Studies focusing on the association of urinary iodine concentration and dietary iodine intake are scarce and generally conclude that women with smaller placental size are more frequently prone to iodine deficiency [55]. A 2011 study comparing Irish and Iranian women, however, established differences between these two populations with respect to the ratio of placental iodine and urinary iodine concentration [46]. Based on this observation, it could be hypothesized that the placenta may act as a thus far unrecognized iodine reservoir designed to ensure the physiological development of the foetus despite fluctuations in dietary iodine.

At the time of data collection, recommended daily iodine intake was generally 50–100 μg/day with 150 μg/day recommended for pregnant women [56]. The recommendation substantially changed over time, with recommended daily intake established at 150 μg/day for the non-pregnant population and 250 μg/day for pregnant or breastfeeding women. While recommended daily iodine intake for the non-pregnant population can be achieved quite easily, most pregnant/breastfeeding women are unable to achieve the recommended levels of iodine in their diet [11]. In this study, we report that estimated iodine intake levels in pregnant women are below the recommended levels both for the early 1990s as well as for 2016, i.e. in line with previous observations conducted on smaller population samples. In a very recent study using the same Central European population, urinary iodine values of over 150 μg/l, i.e. complying with WHO requirements for pregnant women, were only found in 30% of the total of 750 examined Czech pregnant women [57] with the median urinary iodine concentration above 151 μg/l. This implies that no significant improvement in supplementation has taken place over time (our results originating in the early 1990s are in line with very recent observations). Based on our results, the proper management of latent iodine deficiency seems to constitute a major public health issue in the Czech Republic.

### Strengths and limitations

The major limitation of the study is that due to the design of the study (no biological samples were available for this cohort) we were unable to carry out adjustments for urinary iodine. Due to the large number of subjects, it was necessary to use food frequency questionnaires which were not formally calibrated against diet diaries or biomarkers, i.e. a common approach in the early 1990s. Since the FFQ constitutes a methodological instrument designed to measure long-term dietary behaviour, it is prone to several methodological limitations, including a finite list of foods and food groups [58]. Finally, it is possible that due to the retrospective nature of the questionnaire, some memory bias was introduced when compared with a prospective food records-based methodology.

On the other hand, it must be mentioned that this study is the first to investigate dietary iodine intake in relation to the dietary patterns of pregnant women in the transitional period of the early 1990s in Czechoslovakia. Moreover, it also provides insight into the significant relationship between dietary estimated iodine intake and defined adverse birth outcomes. Additional key points of this study include the relatively large sample size of pregnant women and multiple adjustment for confounding factors, although the possibility of residual confounding can never be entirely ruled out. In addition, the observed dietary patterns are consistent with patterns expected in early 1990s Czechoslovakia, which may be demonstrated e.g. by the absence of typical “imported” patterns such as Mediterranean and vegetarian diets not typical at the time.

## Conclusion

This study reveals the possible influence of dietary iodine intake and its effect on placenta size, which influences the dynamics of intrauterine growth. These results contribute to the much-needed debate on iodine intake during the vulnerable child development period. Following adjustment for multiple confounders, including socio-economic status, our study found placental weight to be the only birth outcome significantly associated with iodine intake. While our research indicates that the placenta may be partially capable of compensating for iodine deficiency, more research into mechanistic relationship between iodine accumulation and placental growth is necessary. Based on our results, however, we encourage researchers to use placental weight as a possible interaction parameter with iodine supplementation in future studies.

## Abbreviations

ALSPAC: Avon Longitudinal Study of Pregnancy and Childhood
CoFID: Composition of foods integrated dataset
ELSPAC: The European Longitudinal Study of Pregnancy and Childhood
FFQ: Food frequency questionnaire
GFR: Glomerular filtration rate
HAPIEE: Health, Alcohol and Psychosocial factors In Eastern Europe study
HCG: Human chorionic gonadotropin
ICCIDD: International Council for Control of Iodine Deficiency Disorders
ISCED: International Standard Classification of Education
ISSHP: International Society for the Study of Hypertension in Pregnancy
LAT1: Human L-type amino acid transporter 1
LAT2: Human L-type amino acid transporter 2
MCT10: Specific thyroid hormone cell-membrane transporter 10
MCT8: Specific thyroid hormone cell-membrane transporter 8
OATP1A2: Organic anion-transporting polypeptide 1A2
OATP4A1: Organic anion-transporting polypeptide 4A1
TSH: Thyroid-stimulating hormone
UIC: Urinary iodine concentration
UNICEF: United Nations Children’s Fund
WHO: The World Health Organization

## References

[1] Pearce EN, Lazarus JH, Moreno-Reyes R, Zimmermann MB (2016). Consequences of iodine deficiency and excess in pregnant women: an overview of current knowns and unknowns. Am J Clin Nutr.

[2] Prado EL, Dewey KG (2014). Nutrition and brain development in early life. Nutr Rev.

[3] Zimmermann M, Delange F (2004). Iodine supplementation of pregnant women in Europe: a review and recommendations. Eur J Clin Nutr.

[4] Harding KB, Peña-Rosas JP, Webster AC, Yap CM, Payne BA, Ota E, De-Regil LM. Iodine supplementation for women during the preconception, pregnancy and postpartum period. Cochrane Database Syst Rev. 2017;3:CD011761. 10.1002/14651858.CD011761.pub2 PMC6464647 28260263

[5] Glinoer D (2001). Pregnancy and iodine. Thyroid Off J Am Thyroid Assoc.

[6] Stricker R, Echenard M, Eberhart R, Chevailler M-C, Perez V, Quinn FA (2007). Evaluation of maternal thyroid function during pregnancy: the importance of using gestational age-specific reference intervals. Eur J Endocrinol Eur Fed Endocr Soc.

[7] Andersen SL. Iodine status in pregnant and breastfeeding women: a Danish regional investigation. Dan Med J. 2015;62(5). pii: B5074. 26050837

[8] WHO | Assessment of iodine deficiency disorders and monitoring their elimination [Internet]. WHO. [cited 2017 Jan 7]. Available from: http://www.who.int/nutrition/publications/micronutrients/iodine_deficiency/9789241595827/en/

[9] Alexander EK, Pearce EN, Brent GA, Brown RS, Chen H, Dosiou C (2017). 2017 guidelines of the American Thyroid Association for the diagnosis and Management of Thyroid Disease during Pregnancy and the postpartum. Thyroid Off J Am Thyroid Assoc.

[10] Lazarus J, Brown RS, Daumerie C, Hubalewska-Dydejczyk A, Negro R, Vaidya B (2014). 2014 European thyroid association guidelines for the management of subclinical hypothyroidism in pregnancy and in children. Eur Thyroid J.

[11] Zamrazil V, Bilek R, Cerovska J, Delange F (2004). The elimination of iodine deficiency in the Czech Republic: the steps toward success. Thyroid Off J Am Thyroid Assoc.

[12] Ryšavá L, Kříž J, Kašparová L, Křížová T, Žoltá M, Lisníková P (2016). Iodine supply and iodinuria among the Czech population between the years 1995 and 2016. Vnitr Lek.

[13] Piler P, Kandrnal V, Kukla L, Andrýsková L, Švancara J, Jarkovský J, Dušek L, Pikhart H, Bobák M, Klánová J. Cohort profile: European Longitudinal Study of Pregnancy and Childhood (ELSPAC) in the Czech Republic. Int J Epidemiol. 2017;46(5):1379–1379f. 10.1093/ije/dyw091 PMC5837270 27380795

[14] European longitudinal study of pregnancy and childhood (ELSPAC). Paediatr Perinat Epidemiol. 1989;3:460–9. 10.1111/j.1365-3016.1989.tb00533.x 2587412

[15] Golding J, Pembrey M, Jones R, ALSPAC Study Team (2001). ALSPAC--the Avon longitudinal study of parents and children. I. Study methodology. Paediatr Perinat Epidemiol.

[16] Boylan S, Welch A, Pikhart H, Malyutina S, Pajak A, Kubinova R (2009). Dietary habits in three central and eastern European countries: the HAPIEE study. BMC Public Health.

[17] NutriDatabaze.cz - Databáze složení potravin České republiky [Internet]. [cited 2017 Jan 12]. Available from: http://www.nutridatabaze.cz/

[18] Online potravinová databáza [Internet]. [cited 2017 Jan 12]. Available from: http://www.pbd-online.sk/

[19] Etusivu - Fineli [Internet]. [cited 2017 Jan 12]. Available from: https://fineli.fi/fineli/fi/index

[20] Danish Food Composition Databank / Database [Internet]. [cited 2017 Jan 12]. Available from: http://frida.fooddata.dk/?lang=en.

[21] Composition of foods integrated dataset (CoFID) - Publications - GOV.UK [Internet]. [cited 2017 Jan 12]. Available from: https://www.gov.uk/government/publications/composition-of-foods-integrated-dataset-cofid

[22] Mullerova D, Kopecky J, Matejkova D, Muller L, Rosmus J, Racek J (2008). Negative association between plasma levels of adiponectin and polychlorinated biphenyl 153 in obese women under non-energy-restrictive regime. Int J Obes.

[23] Český statistický úřad | ČSÚ [Internet]. [cited 2017 Jan 12]. Available from: https://www.czso.cz/

[24] Dofkova M, Kopriva V, Resova D, Rehurkova I, Ruprich J (2001). The development of food consumption in the Czech Republic after 1989. Public Health Nutr.

[25] Macdonald-Wallis C, Silverwood RJ, de Stavola BL, Inskip H, Cooper C, Godfrey KM, et al. Antenatal blood pressure for prediction of pre-eclampsia, preterm birth, and small for gestational age babies: development and validation in two general population cohorts. BMJ. 2015;351:h5948. 10.1136/bmj.h5948. 10.1136/bmj.h5948 PMC4647185 26578347

[26] Choi H, Rauh V, Garfinkel R, Tu Y, Perera FP (2008). Prenatal exposure to airborne polycyclic aromatic hydrocarbons and risk of intrauterine growth restriction. Environ Health Perspect.

[27] Median income - Eurostat [Internet]. [cited 2017 Jan 12]. Available from: http://ec.europa.eu/eurostat/en/web/products-statistical-reports/-/KS-FT-17-004.

[28] Hair JF, Anderson RE, Tatham RL, Black WC (1995). Multivariate data analysis (4th Ed.): with readings.

[29] Mardia KV, Kent JT, Bibby JM. Multivariate analysis / K.V. Mardia, J.T. Kent, J.M. Bibby [Internet]. London ; New York: Academic Press; 1979 [cited 2015 Feb 6]. Available from: https://catalog.loc.gov/vwebv/search?searchCode=LCCN&searchArg=79040922&searchType=1&permalink=y.

[30] Multiple Imputation by Chained Equations (MICE): Implementation in Stata | Royston | Journal of Statistical Software [Internet]. [cited 2016 Mar 16]. Available from: https://www.jstatsoft.org/article/view/v045i04

[31] Venkaiah K, Brahmam GNV, Vijayaraghavan K (2011). Application of factor analysis to identify dietary patterns and use of factor scores to study their relationship with nutritional status of adult rural populations. J Health Popul Nutr.

[32] Bienertová-Vašků J, Zlámal F, Pruša T, Novák J, Mikeš O, Čupr P, et al. Parental heights and maternal education as predictors of length/height of children at birth, age 3 and 19 years, independently on diet: the ELSPAC study. Eur J Clin Nutr. 2017;71(10):1193–1199. 10.1038/ejcn.2016.244 28176773

[33] National Institute for Health and Welfare. National Food Composition Database in Finland [Internet]. [cited 2016 May 5]. Available from: https://fineli.fi/fineli/en/index?

[34] DTU Fødevareinstituttet. Fødevaredata version 1 | udgave 2015–12-04 [Internet]. 2016 [cited 2016 May 5]. Available from: http://frida.fooddata.dk/index.php?lang=en

[35] Státní zdravotní ústav. Zásobení jódem jako prevence tyreopatií - sborník [Internet]. SZÚ; 2016. Available from: www.szu.cz/konference-ke-dni-jodu

[36] Bliddal S, Boas M, Hilsted L, Friis-Hansen L, Tabor A, Feldt-Rasmussen U (2015). Thyroid function and autoimmunity in Danish pregnant women after an iodine fortification program and associations with obstetric outcomes. Eur J Endocrinol.

[37] Bath SC, Steer CD, Golding J, Emmett P, Rayman MP (2013). Effect of inadequate iodine status in UK pregnant women on cognitive outcomes in their children: results from the Avon longitudinal study of parents and children (ALSPAC). Lancet Lond Engl.

[38] Hynes KL, Otahal P, Hay I, Burgess JR (2013). Mild iodine deficiency during pregnancy is associated with reduced educational outcomes in the offspring: 9-year follow-up of the gestational iodine cohort. J Clin Endocrinol Metab.

[39] Abel MH, Caspersen IH, Meltzer HM, Haugen M, Brandlistuen RE, Aase H (2017). Suboptimal maternal iodine intake is associated with impaired child neurodevelopment at 3 years of age in the Norwegian mother and child cohort study. J Nutr.

[40] Yang XF, Xu J, Hou XH, Guo HL, Hao LP, Yao P (2006). Developmental toxic effects of chronic exposure to high doses of iodine in the mouse. Reprod Toxicol.

[41] Brown RS (2004). Minireview: developmental regulation of thyrotropin receptor gene expression in the fetal and newborn thyroid. Endocrinology.

[42] Matsuo H, Maruo T, Murata K, Mochizuki M (1993). Human early placental trophoblasts produce an epidermal growth factor-like substance in synergy with thyroid hormone. Acta Endocrinol.

[43] Costa A, Arisio R, Benedetto C, Bertino E, Fabris C, Giraudi G (1991). Thyroid hormones in tissues from human embryos and fetuses. J Endocrinol Investig.

[44] Loubière LS, Vasilopoulou E, Bulmer JN, Taylor PM, Stieger B, Verrey F (2010). Expression of thyroid hormone transporters in the human placenta and changes associated with intrauterine growth restriction. Placenta.

[45] Burns R, O’Herlihy C, Smyth PPA (2011). The placenta as a compensatory iodine storage organ. Thyroid Off J Am Thyroid Assoc.

[46] Burns R, Azizi F, Hedayati M, Mirmiran P, O’Herlihy C, Smyth PPA (2011). Is placental iodine content related to dietary iodine intake?. Clin Endocrinol.

[47] Roland MCP, Friis CM, Voldner N, Godang K, Bollerslev J, Haugen G (2012). Fetal growth versus birthweight: the role of placenta versus other determinants. PLoS One.

[48] Barker DJP, Thornburg KL, Osmond C, Kajantie E, Eriksson JG (2010). The surface area of the placenta and hypertension in the offspring in later life. Int J Dev Biol.

[49] McCrabb GJ, Egan AR, Hosking BJ, ALEXANDER G, Allden W, Barcroft J (1992). Maternal undernutrition during mid-pregnancy in sheep: variable effects on placental growth. J Agric Sci.

[50] Hutcheon JA, McNamara H, Platt RW, Benjamin A, Kramer MS (2012). Placental weight for gestational age and adverse perinatal outcomes. Obstet Gynecol.

[51] Eriksson J, Forsén T, Tuomilehto J, Osmond C, Barker D (2000). Fetal and childhood growth and hypertension in adult life. Hypertension.

[52] Holroyd CR, Osmond C, Barker DJ, Ring SM, Lawlor DA, Tobias JH (2016). Placental size is associated differentially with postnatal bone size and density. J Bone Miner Res Off J Am Soc Bone Miner Res.

[53] Villar J, Papageorghiou AT, Pang R, Ohuma EO, Cheikh Ismail L, Barros FC (2014). The likeness of fetal growth and newborn size across non-isolated populations in the INTERGROWTH-21st project: the fetal growth longitudinal study and newborn cross-sectional study. Lancet Diabetes Endocrinol.

[54] Rasmussen LB, Ovesen L, Bülow I, Jørgensen T, Knudsen N, Laurberg P (2002). Dietary iodine intake and urinary iodine excretion in a Danish population: effect of geography, supplements and food choice. Br J Nutr.

[55] Olivares JL, Olivi GI, Verdasco C, Ortiz VA, Mayer MA, Cresto JC (2012). Low iodine intake during pregnancy: relationship to placental development and head circumference in newborn. Endocrinol. Nutr. Órgano soc. Esp. Endocrinol. Nutr.

[56] Delange F (1994). The disorders induced by iodine deficiency. Thyroid Off J Am Thyroid Assoc.

[57] Bílek R, Kaňová N, Mindžáková V, Neumann D, Jiskra J, Ryšavá L (2016). Iodine supply of pregnant women in the Czech Republic. Vnitr Lek.

[58] Crume TL, Brinton JT, Shapiro A, Kaar J, Glueck DH, Siega-Riz AM, et al. Maternal dietary intake during pregnancy and offspring body composition: The Healthy Start Study. Am J Obstet Gynecol. 2016;215(5):609.e1–609.e8. 10.1016/j.ajog.2016.06.035 PMC5571832 27371352

